# Principles of Pathology, &c.

**Published:** 1837-01

**Authors:** 


### Art. V.

1. *Principles of Pathology, and Practice of Physic.* By John Mackintosh, m.d., Lecturer on the Practice of Physic in Edinburgh, &c. *Fourth Edition.*—Two vols. 8vo. pp. 524, 546. *London,* 1836.

2. *Principles of Pathology, and Practice of Physic.* By John Mackintosh, m.d. &c. *From the last London (Third) Edition, with Notes and Additions.* By S. G. Morton, m.d. &c. &c.—Two vols. 8vo. pp. 462, 509. *Philadelphia,* 1836.

We received, nearly about the same time, the two books of which we have transcribed the titles; and it certainly speaks favorably for any work when a *third* edition of it, republished in a foreign country, is met by one still newer on its reaching the mother-land of both. The volumes before us are unquestionably not common ones, or we should not have thought it necessary to notice them further than to announce their general character; as, of all publications, new editions of systematic treatises generally present the least matter for interesting analysis or criticism. But it is impossible to inspect any portion of Dr. Mackintosh's book without being at once convinced that it is very dissimilar to the ordinary systematic treatises on medicine, and that the author possesses but a scanty share of the qualities of an ordinary compiler. In truth, although bearing the outward and visible signs of a compendium of the Practice of Physic for the industrious student, it has very little of the sustained soberness and dull gravity supposed to characterize such collections; but a vast deal of the raciness of originality, and not a slight tincture of the waywardness of genius. Judging from his book alone, we should have considered Dr. Mackintosh the least likely person in the world to meditate the plan of a formal treatise on Medicine, or to submit to the drudgery of filling up its details; but we suppose the necessities of the teacher rather than the natural predilections of the physician gave occasion to the work, in the first place; and, although the favour of the public has gradually swelled it from the humble original form of "Heads of Lectures" into the comprehensive "System of Pathology and Practice of Physic," which is now before us, it still bears characteristic marks of its origin, and of being the production of a mind hardly suited to the humble task it had undertaken. We can fancy how admirably the licence of oral instruction would harmonise with the freedom and boldness and occasional vehemence of the author, while he seems cabin'd, cribbed, confined, by the requisites of written composition. These qualities of the writer necessarily confer on the work the character of inequality, both as to extent and variety of the matter contained in different parts of it, and the manner in which this is worked up. Many chapters in it are certainly extremely meager and unsatisfactory, and quite unsuited to direct the student in the cases of which they treat; and others are devoted rather to the exposition of some peculiar views of the author, than to the elucidation of the best established principles of pathology, or the inculcation of the soundest practice. In a good many cases, also, Dr. Mackintosh has failed to lay before the reader the latest pathological facts, or to notice modes of treatment resting on the highest authority; and, still more frequently has he contented himself with illustrating his subjects by reference to a few works of domestic origin and of not very recent date, when he might have been supplied with much more copious and striking illustrations from other authors of more extensive experience and higher authority. Still, with all these defects, and perhaps with some others both of matter and manner, we feel it our duty to admit, with the American editor, "the decided superiority of Dr. Mackintosh's Pathology and Practice of Physic over almost all works of a similar character," that is to say, all the systematic works hitherto produced in this country as manuals for students and junior practitioners. The whole form and spirit of the work is essentially pathological and scientific, and in direct opposition to the empirical plan so often adopted in publications of a similar class; it wages relentless war with old prejudices and obsolete theories, and, we may truly say, with all their sectators and abettors dead and living; it everywhere inculcates the most active and the boldest treatment; it loses no opportunity of holding up to honour and praise the excellence and dignity of our art; and, fraught as it is throughout with the spirit of its author, it is especially calculated to communicate to the youthful mind that most precious of all lessons, enthusiasm in the study and practice, and confidence in the powers of medicine.

We should have been glad if the work of Dr. Mackintosh had found in the American Editor a more mechanical and book-learned foster-father, who might have supplied from his own stores or from the works of others the deficiencies in the original of which we have complained. This has, however, been but very imperfectly done, although a good deal of valuable matter has been added in some places which Dr. Mackintosh will do well to retain in his next edition. But much more is wanting to make the work a complete Introduction to the practice of physic; and we earnestly advise the author, when another edition is called for (as doubtless will be the case ere long,) to take effectual measures to have the defects remedied and the omissions supplied. All that would seem necessary for this purpose is for the author, or, what would probably be still better, some more bookish and less busy friend, to read carefully the standard systematic works and monographs on different diseases published in France, Germany, and our own country, during the last ten years, and transfer to his pages such pathological and practical facts as would at once render the different parts of the work of more equal proportions, and bring down the state of knowledge to the present day in those instances where it lags behind. By way of instancing the deficiencies alluded to, we may state that we do not observe in these volumes any reference to the *three* most recent French Dictionaries, nor to such important pathological works as those of Louis, Chomel, Bouillaud, Piorry, Cruveilhier, and others among the French; and to no recent German author; neither do we find once noticed the splendid and invaluable work on morbid anatomy by Dr. Carswell, nor Dr. Copeland's Dictionary, nor even the Cyclopædia of Practical Medicine; although if any work can be considered as embodying the present state of medical science in this country, it is surely that elaborate and learned collection.

We will now, without attempting to criticise, much less to analyse the whole work, notice a few subjects in it here and there, which we noted as most deserving the reader's attention, whether for their novelty, singularity, or practical importance. We should be sorry if our extracts prevented any of our readers from making themselves masters of the original, which is calculated to gratify and benefit the man of experience no less than the student.

The first part of Dr. Mackintosh's work treats of "the general history of inflammation and fever, with the pathology and treatment of individual fevers;" and, although from the nature of the subject necessarily containing a good deal that is doubtful and questionable in doctrine, is characterised throughout by sound and what may be called common-sense views of pathology, and by the inculcation of the most rational and vigorous practice. Like all exercised and sober-minded physicians of the present day, in his views of the nature and treatment of fevers, Dr. Mackintosh is an eclectic, and, in accordance with the cool and practical spirit of British medicine, never hesitates to lay down his theories at the command of experience. Without being a Broussaian, he adopts all the valuable improvements introduced by Broussais into the treatment of fever, while he repudiates all fantastical and feeble practice, from whatsoever authority it may emanate or whether countenanced by theory or not. "The best axiom in physic," says Dr. Mackintosh, "is, to get rid of diseased action as quickly as possible, as there is no saying what mischief is to follow in the train of consequences." In the American Edition an elaborate exposition of the Broussaian or physiological Doctrine of Fever is introduced, on the principle, as stated by the editor, that "however much medical men may differ in opinion respecting this theory, there is an obvious propriety of its being understood by every member of the profession."

Agreeing with Dr. Fordyce in his often-quoted opinion of the universality of fever, Dr. Mackintosh gives the following propositions as embodying his own opinions and as containing "general views which are admitted by all writers whose opinions are of any value, although the same facts have been called by different names."

"1st. That the functions of almost all organs are embarrassed in fever from the very beginning, and often for days before the sense of coldness is felt by the affected person. 2d. That the blood leaves the surface of the body, and accumulates in internal organs, and that, unless they are overwhelmed, the system makes an effort to relieve herself, and certain combined phenomena take place, which are designated by the terms 'reaction, fever.' 3d. That inflammation of all parts of the body will give rise to fever. 4th. That inflammation may supervene during fever, without being the primary cause of the febrile commotion. 5th. That the nervous system is involved as well as the vascular; and, 6th, It follows as a consequence, if all these things be true, that the blood itself must be in a diseased condition." (P. 35.)

The following is his classification of fevers, which, although open to obvious objections, enables the author to arrange his facts conveniently. The most palpable defect is that which classes the gastric fever of children, usually termed *Infantile remittent*, with the remittent fevers of climatorial or local origin, and the yellow fever as essentially belonging to this class, when it is well known that the disease so termed, although occasionally strictly a remittent fever, is often of quite a different character.

"1st. Intermittent Fever.
"2d. Remittent or Yellow Fever: Infantile Remittent.
"3d. Continued Fever, subdivided into four orders, viz.
Fever from functional derangement.
—— from inflammation.

Fever from congestion.
A mixed form of fever between these three last, but in which congestion predominates, commonly denominated Typhus or Synochus.
"4th. Hectic fever.
"5th. Fevers attended with eruptions.
"6th. The Plague." (P. 37.)

The peculiar practice of bleeding in the cold stage of intermittent fever, introduced by Dr. Mackintosh a good many years since, is well known to most of our readers. This practice naturally takes a prominent place in the chapter treating of ague, and is illustrated and supported by many new cases. Without admitting that such treatment is necessary in the great majority of cases of ague, as we know from considerable experience that the disease is perfectly and safely and speedily curable without it, we cannot deny that the practice is in particular cases indispensable, and indeed affords the only means of saving life. We must therefore always consider medical science as under great obligations to Dr. Mackintosh for having introduced this practice, and for having advocated it with such praise-worthy zeal. We are far from wishing our younger brethren to have recourse to it in ordinary cases, much less habitually, in the treatment of ague; as we are of opinion that this would be not merely useless but highly injurious; but it is incumbent on every one to make himself acquainted with the mode of its application, the principles on which it is founded, and the safety of its administration. For our own parts, we do not hesitate to say that, had we been acquainted with this practice twenty years ago, we think we could have saved some lives which we allowed to be hopelessly lost beneath the overwhelming stupor of inter-tropical ague.

The following brief extracts exhibit the pathological grounds on which Dr. Mackintosh was led to employ this remedy, and the general mode and limits of its application; but we earnestly recommend to the notice of our readers the whole chapter on this subject, and the numerous cases by which the practice is illustrated and confirmed.

" *Cold Stage*. The first circumstance which we distinctly perceive, is diminished circulation of blood in the extremities, then a sense of coldness, and with it a feeling of weakness. These are evidences of an irregular determination of blood, by whatever cause produced; and in proportion as blood accumulates in the vessels of internal organs, their functions become impeded. The lungs shew their gorged state, by the short, difficult, and anxious breathing; by the impossibilty of inflating them beyond the least degree; and by the violent dry cough which occasionally takes place. The livid appearance of the cheeks, lips, and mucous membrane of the mouth, is an additional proof of the embarrassed state of the lungs, shewing that the blood is not properly decarbonized. The disordered functions of the brain in this stage, depend, I imagine, principally upon the gorged state of the lungs, and also upon the overloaded state of the right side of the heart, preventing the free return of blood from the head. The disordered functions of the brain may also be produced by a change in the balance of the circulation of the vessels of the head, independently of the state of the lungs and heart. The tremors may probably be attributed to an accumulation of blood in the vessels of the brain and spinal marrow. The sense of cold seems to be owing partly to the state of the nervous system, and partly to the state of the lungs. The pain in the head and loins, and oppression at the præcordia, may be fairly attributed to the same causes. The muscular prostration, and feeling of sinking, are not owing to actual debility, but to obstructed action, in consequence of the above-mentioned condition of organs. The proof of all which circumstances is to be found in the fact, now well known, that abstracting blood, in the cold stage, will immediately remove not only the difficulty of breathing, the pain in the head and loins, the disordered functions of the brain, (when these exist,) the oppression at the præcordia, &c. but will also stop the rigors, restore the strength of the pulse, increase the heat of the whole body, and cause the sensation of cold to vanish in an instant." (P. 80.)

"Bleeding, in the cold stage, will, in a great majority of instances, cut it short; in fact, it will rarely fail in stopping the existing paroxysm, and, on many occasions, it has prevented a return of the disease to which the patients had been long subject, and by which they were nearly worn out. It is difficult to determine what quantity of blood it will be necessary to draw in any given case; sometimes it requires twenty-four ounces; I have known three ounces suffice, and, in one case, an ounce and a half produced the full effect. The larger the orifice in the vein, the greater is the chance of arresting the disease at a small expense of blood; but, in many cases, the operation is attended with considerable difficulty, from the convulsive tremors which affect the whole body. I was once successful in arresting the disease by bleeding, in a cold stage which had continued twenty-six hours; but I regard this as an extreme case. The blood sometimes only trickles down the arm, and, as the system is relieved, the stream becomes larger and stronger, till at last it springs from the orifice, and frequently before six ounces are taken, the patient will express relief from violent pain in the head and loins, and it will soon be observed that he breathes more freely. The tremors become slighter and slighter, and, by the time a few more ounces are abstracted, they will cease altogether, and with them will vanish the painful sensation of cold. The pulse will be found stronger, and a gentle moisture will be observed on the body. If the patient be properly managed with respect to bed-clothes, neither the hot nor sweating stage will in general follow. Most of the patients who have been treated by myself, or by my pupils under my immediate inspection, have fallen asleep immediately after the operation; but some have even got up and dressed themselves." (P. 86.)

Of course, Dr. Mackintosh employs the ordinary means during the interval for preventing the return of the paroxysm. His mode of prescribing quinine "is to give three doses of five grains each, with half an hour of interval, immediately before the expected paroxysm; as, three grains every half hour, beginning about three hours before the expected paroxysm." (I. p. 130.) Our own experience leads us to recommend the practice of the American editor, rather than that of Dr. Mackintosh; a practice which we have scarcely ever found to fail, even with patients continuing to reside in the malarious locality which produced the disease.

"We would wish to be understood," (says Dr. Morton,) "as not according with the practice of administering quinine in the large and frequently repeated doses advocated by the author. In this country, it is seldom requisite to administer more than twelve grains during the first interval, and half that quantity during the following interval, to cure nearly all the cases which occur. Instances, however, frequently present themselves in which the exhibition of a larger quantity than is necessary to attain the end, would be positively injurious; so that practitioners have adopted the safer plan of giving a grain each hour, and limiting the amount to the number of grains specified." (Vol. I., p. 131.)

We even find that half the quantity above stated, or one grain every two hours, is quite sufficient in the majority of cases to cure the endemic agues of England.

The following interesting note on the etiological relations of intermitting fever is extracted from the additions in the American edition: it originates from the French school.

"M. Brachet has paid some attention to the phenomena of intermittents. Basing his theory upon the peculiar views which he takes of the offices and connexions of the two nervous systems, the cerebro-spinal and ganglionic, he attributes the primary lesion to their derangement, to the exclusion of irritation as understood by the physiological school. The ganglionic system presides over all the actions of organic life, as nutrition, secretion, &c. while the nervous system of relation has charge of the connexion with the exterior world. According to him, the phenomena of intermittent fever are such as can only be produced by derangement of the healthful influence of the first, communicated to the second ; and no matter whether the modifying impression is made externally by atmospheric or physical agents, or takes place internally by marsh miasm; the first effect is produced on the nerves of the organic movements. That the result of this impression is not inflammation, he proves by the following experiments. Towards the end of October, 1822, he took for seven nights in succession, at midnight, a cold bath in the river Saone. The first bath was of a quarter of an hour's continuance; the second half an hour ; from this he went on protracting the time, until he was enabled to remain in the water a whole hour. After each bath, he laid [lay] down in a warm bed and underwent considerable reaction, with increased warmth, followed by profuse sweating; after which he went to sleep. At the expiration of seven days, M. Brachet omitted his experiments, but was surprised to find, during the following day, that between twelve and one o'clock, p. m. all the attendants of a true intermittent paroxysm made their appearance. As he experienced no inconvenience during the interval, he allowed this artificial fever to proceed, and experienced six distinct attacks. Upon the seventh night following the last bath, he was called upon to ride some distance upon professional business, a short time prior to the expected invasion; the exercise thus taken produced excitement of his system, which was kept up by placing himself near a large fire, and from that time no accession reappeared. This account corroborates, in a measure, the statement of M. Roche that intermittence of cause will produce a habit, more or less difficult to counteract, in proportion to the fixedness of it. In speaking of these conclusions of M. Brachet, it is understood, that the paroxysm is simple in character, unaffected by organic lesions, which will modify its type and be productive of such complications as are found in these fevers of serious grade." (P. 103.)

The Article on Continued Fever is full of excellent matter pathological and practical. We regret much that want of space prevents us from making any extracts from it.

The author seems less at home on the subject of Yellow Fever; but the chapter contains many interesting details, more particularly those tending to prove the non-contagious nature of the disease as it has prevailed of late years in the south of Spain and in our garrison of Gibraltar. To this part of the work the American editor has made some valuable additions chiefly on the morbid anatomy of the disease as seen of late years in America. The dissections here recorded tend strongly to support the view of the gastric origin of the disease, first advanced, we believe, by Tommasini and the other Italian physicians in their account of the fever of Leghorn in 1804.

The following observations from the same chapter prove that the mercurial plan of treating yellow fever, formerly so prevalent in America as elsewhere, has given way to a more rational system, at least; whether to one more successful in its results, remains probably yet to be ascertained. Dr. Morton is so obviously a follower of Broussais, that his authority may be somewhat questionable, without any impeachment of his honesty.

"It is at least evident, that the exhibition of the enormous quantities of mercury which have been given both in this disease, and in other forms of fever, is not attended with the unfailing success which alone could warrant its employment; and the after consequences are so frequently destructive to health and comfort, that the opposite extreme of total proscription of this powerful article of the materia medica has been fallen into; a circumstance much to be deplored, as in proper doses and at suitable periods of the affection, its use is highly serviceable. The gastric character of yellow fever appears at the present era to be well ascertained; and the clinical reports of those who have treated it in accordance with the pathological doctrines which are becoming every day more widely disseminated, afford evidence of the superior utility of strictly antiphlogistic measures. If the general system is affected with considerable reaction, venesection is required; but in most cases the prompt application of leeches, or cups, [cupping glasses] as near as possible to the diseased organs, is followed by a decided amelioration of the symptoms. They should not be placed, however, so immediately in contact as to run the risk of increasing excitement. As an auxiliary measure, the sedative impression of cold has a beneficial effect, and is peculiarly grateful to the patient: iced drinks, ice applied to the head, if this organ presents symptoms of disordered action, and the injection of cooling enemata into the bowels, are the modes of application. The administration of small doses of calomel, or blue pill, will admirably promote the cure when the force of the local irritation has been reduced; and it only remains to unlock the secretions and gradually lead them back to a natural state." (P. 147.)

There is no part of his work in which Dr. Mackintosh shows a greater spirit of innovation, or more disregard of ancient views and practice, than in his account of Eruptive Fevers. Here he evidently has taken Broussais for his guide, but he has superadded all his own therapeutic boldness to complete a picture which cannot fail to have a strong influence on the inexperienced mind. We think it therefore right to interpose a word of caution to those who shall proceed for the first time to treat eruptive fevers upon the principles here laid down. We differ very little from Dr. Mackintosh either in his views or practice; but we believe that a good deal of discrimination is necessary in selecting the cases to which the latter is most applicable. Subjects previously debilitated or labouring under depression or irritation of the nervous system, whencesoever derived, will not always bear the *medicina perturbatrix* here advocated; but we have no manner of doubt that, applied to epidemics in country places, in the army and navy, and indeed generally to individuals previously in good health and without any constitutional idiosyncracies, it will be the means of saving innumerable lives. We can only find room for a couple of extracts explanatory of his pathological views and practice in such cases. Although the latter is, he conceives, supported by the former, he wishes to found its claim to general adoption on the more solid basis of experience.

"After a long and patient investigation, comparing the symptoms with the appearances found on dissection, I have come to the opinion, that the mucous membranes are the seat of the disease, the nature of which is inflammation, more or less acute and extensive; and that the part generally most implicated, is the mucous membrane of the lungs, particularly in measles and small-pox; while that of the bowels is the part chiefly, if not principally, affected in urticaria, roseola, and miliary fever. The eruption is merely to be regarded as a symptom, and by no means a universal symptom. It is well known that many cases of eruptive fevers are very mild, and require little treatment, while others are extremely severe and fatal; and that a great deal depends upon the eruption, whether it comes out at the usual period, and whether it remains out, or prematurely and suddenly recedes. The eruption, in point of fact, ought to be regarded as a natural blister, acting as a contra-irritant." . . . . "These circumstances, it appears to me, are clearly proved, 1. By attending to the constitutional commotion and oppression of the whole system, and the morbid changes in the functions of various organs, for many days before the appearance of the eruption. 2. By the relief afforded, in general, after the free development of the eruption. 3. By the increased suffering and danger which exist when the eruption is deficient, or when its repulsion suddenly and prematurely takes place. 4. By the relief which follows proper treatment; and, 5, by the appearances observed on dissection." (Vol. i. p. 177.)

The following brief extracts show the application of this practice to the chief eruptive fevers.

"If these observations be not fallacious, bleeding to a sufficient extent ought not only to relieve the constitutional symptoms during the eruptive fever, but after the eruption has appeared, ought to destroy it. Observations and experiments frequently performed and repeated by myself, and by my pupils, enable me to state, that these are facts, which I shall not be afraid to repeat before the highest authorities in the profession, and stake my professional reputation upon the general result of the plan; having already seen recoveries take place, under this treatment, in cases in which such a happy termination was scarcely to be anticipated. It also follows, if these things be true, that even in ordinary cases there are two periods more critical and dangerous to the patient than any other; these are, the period at which the eruption ought to make its appearance, and that at which it should naturally disappear. In the first case, the internal disease has gradually become extensive and severe, and wants relief by means of the eruption. In the second, the disease which had existed at first, having been relieved by the external irritation, is now in danger of being reproduced by its cessation; and this of all others is the period at which, in the slightest form of the disease, the patient stands most in need of care and vigilant attention to the condition of internal organs." (Vol. i. p. 183.)

*Scarlet Fever.* "In the slighter forms of scarlatina, very little treatment is necessary, further than confinement, attention to the bowels to keep them free, and the antiphlogistic regimen. In such cases, however, the medical attendant should be careful to watch diseased action, at the period when the eruption naturally declines, for reasons already mentioned. Formerly I saw many fatal cases of scarlatina, when I practised according to the opinion of the schools, carefully abstaining from blood-letting, and using all the means recommended to support the strength; but I occasionally observed patients snatched from the grave by considerable bleedings from the nose, and at times when it was thought the loss of an ounce of blood would prove destructive. These circumstances, together with the appearances found on dissection, led me to bleed in many subsequent cases, and I have never had occasion to regret it. Blood has been drawn at all periods of the disease, in cases where the state of the lungs and brain required it; and should the operation be performed during the period of the eruption, it will disappear, if a sufficient quantity of blood be taken, When the inflammation of the throat runs very high, I know no remedy productive of such certain and immediate good effects as general bleeding, but should the patient's strength be already reduced, leeches are to be preferred." (Vol. i. p. 189.)

Dr. Morton informs us that the profession in the United States is divided in opinion as to the use of venesection in Scarlatina, but that he himself has resorted to it in every case, and with the most gratifying results. "It is almost in vain (he says,) to treat the congestive form in any other way; and, in the violently inflammatory disease, there is no substitute for the lancet." (Vol. I., p. 195.)

*Measles.* "In the slighter forms of this disease, as in scarlatina, very little treatment is necessary, further than confinement to one room, the exhibition of gentle laxatives, and low diet. The medical attendant should be still more watchful in this disease than in scarlet fever, at the period when the eruption naturally recedes. In the severer forms of measles, bleeding is often necessary during the eruptive fever, when the pectoral symptoms run high, and appear threatening; and also when coma and convulsions take place, both of which are more likely to happen, but particularly the latter, if the child be suffering from difficult dentition." . . . . "When bleeding is necessary, it ought to be performed in the manner already described when treating of inflammatory fever; a sufficient quantity should be taken as early as possible in the disease, and the operation ought to be repeated at short intervals; but when the bronchitic symptoms have been allowed to go on neglected till the air passages are gorged with mucus, bleeding is a very questionable remedy, and no doubt often does irreparable mischief." (P. 198.)

*Small-Pox.* "Bleeding has been often employed, and strongly recommended in this disease, particularly during the eruptive fever; but it has as often been condemned, because it has destroyed that strength which, it is alleged, is so much required in the latter stages of the diseases. But the same language is used in the purest inflammatory fevers. In all the successful cases of confluent small-pox occurring in adults which I have treated, except one, (amounting in all to about eighteen,) bleeding was employed, and largely employed, in the eruptive fever, to moderate what was thought to be local inflammation, without suspecting that they were cases of small-pox: several of the sufferers were my pupils, who had had themselves bled before they sent for me. In a number of instances, blood has been drawn even after the appearance of the eruption, and with decided benefit; but, upon the whole, it is perhaps best at that period to trust to leeches for relieving local inflammations." (P. 204.)

According to the author's views of the nature of the disease, this is the class of affections among which *Erysipelas* should have been placed, and not among the chronic diseases of the skin, to which it has scarcely any other relation than that of locality. Its pathology, according to Dr. Mackintosh, is almost identical with that of eruptive fevers, and its treatment is similar. His observations on this disease are particularly worthy of notice.

"It is truly lamentable," he says, "to reflect how fatal erysipelas has always been, and continues to be, not only in public hospitals but in private practice. . . . Many of my medical acquaintances are as much afraid of erysipelas as they would be of the plague; others, from the dread of typhoid symptoms, and of mortification and putridity, aggravate the disease by improper remedies. . . . How are these bad consequences to be prevented in subsequent cases? The answer is easy, and the practice simple, provided medical men would use the common sense with which they are endowed, and give up a prejudice that has been inculcated on their minds from the earliest period of their lives,—forgetting that there is any thing mysterious in erysipelas,—and learning to treat each case that comes before them upon its own individual merits." (Vol. ii. p. 266.)

The remedies on which Dr. Mackintosh places his chief dependence are those found beneficial in other inflammations, viz. general bleedings in the early stage, followed up by the application of leeches to the inflamed part, purgatives, antimonials, opiates, &c.

The second part of Dr. Mackintosh's treatise comprehends the "Diseases of the Organs connected with the Digestive System;" that is, all the diseases having their site in the abdomen and its outlets, whatever be their nature. The only one of this interesting class of affections which our limits permit us to notice is the Asiatic cholera, of which disease an account is given for the first time as it was witnessed by the author during the presence of the epidemic in Edinburgh. We regret much that we can only notice this chapter very briefly, as it possesses, on many accounts, particular interest.

Dr. Mackintosh's opportunities for witnessing the disease were most ample, and he appears to have devoted himself to its investigation and treatment with all the energy of his character. Into the Drummond-street Cholera Hospital, of which Dr. M. was physician, there were received 461 patients, of which number 291 died; and of these no less than 280 were examined most minutely after death; the greater number by Dr. Mackintosh's own hands, and each examination occupying generally two hours.

At the commencement of the disease, Dr. M. was a decided believer in the contagious nature of cholera; but this belief was greatly weakened by what he witnessed and underwent during the epidemic; his opinion now being "that, if it be contagious, it is not so in any very great degree." Indeed, after some of the details supplied by Dr. M. in reference to this point, we are rather surprised that he admits its contagiousness in any degree. He himself, as well as the young medical officers, seem almost to have lived in the hospital, going to sleep from exhaustion on the very beds of the dying and the dead. A few nurses had the disease, but not severely, and almost all those attacked were persons addicted to drinking.

"None of the house-surgeons, the number being between twenty and thirty, who were seldom out of the wards, had the disease, although their bodies must have been ready to receive the contagion, if fatigue of body, anxiety of mind, and want of sleep, ever predisposed any person to take a disease." (Vol. i. p. 344.)

"In the Drummond-street Cholera Hospital there were 280 bodies examined. Two, and sometimes three hours, were spent in examining each body. From the economical arrangements of the Board of Health, and the difficulty of procuring a proper apartment, the dead-room, where these examinations were conducted, was a miserable place about eight feet square; generally six or eight persons were present, sometimes more; and in an inner apartment, about ten feet square, there sometimes lay six dead bodies. Not one of those who frequented this den of death, and who had their hands imbrued in the secretions of the dead for six hours out of the twenty-four, were affected with cholera, although their hands were irritated and punctured daily!" (P. 345.)

None of the arguments in favour of the epidemic nature, and against the contagion of cholera, appear to us more striking than the fact that *animals* suffered in various places where the epidemic was raging. This was particularly remarked in Austria, as appears by a report of the Medical Faculty of Vienna, of which notice is taken in another part of our present Number. Similar phenomena were witnessed during the prevalence of the disease in Scotland.

"Mr. Dick, the professor of veterinary medicine in Edinburgh, published a paper in the Veterinarian for April, 1833, wherein it is shown that cholera was by no means uncommon among domestic animals, particularly horses and cows, during the epidemic season in Edinburgh. They had diarrhœa and rigid cramps; the blood was viscid and dark; the discharge from the bowels resembled that from the human subject. Several animals died suddenly, and the appearances on dissection resembled those in the human subject, particularly in the stomach and bowels." (P. 345.)

The appearances observed on dissection are minutely given, and are extremely interesting. We can only here notice a few of the most prominent. Dr. M. most properly separates the morbid appearances which there was reason to believe unconnected with cholera from those that unquestionably were so; and also distinguishes the appearances observed in persons dying in the stage of collapse, from those found in such as survived this, and fell victims to the subsequent fever. The following is the condition of the blood and of its distribution in persons who died in the collapsed stage, and is extremely striking:—

"The blood attracted our attention in the first dissection, and it had the same appearances to the last. It was dark coloured, and had lost much of its fluidity:

this was expected, from the accounts that had previously reached us from other countries. But we were astonished to find that it was contained in the arteries and veins, in the most minute capillary, as well as in the larger vessels; that it had the same dark colour in both sets of vessels, some of them containing a small quantity, others being enormously distended. The capillaries and large veins on the surface of the body contained as much blood after death as during life. On opening a vein in the dead body, the blood flowed almost as readily as it had done during life in the same person. The surface therefore retained the same dark appearance as it presented during life, and the muscles were of a dark red colour. In the act of death, or immediately afterwards, in all other diseases, the blood leaves the capillaries, recedes from the surface, and collects in the heart and large veins near it; the arterial system is generally quite empty, but occasionally a little blood is found in the aorta." (Vol. i. p. 347.)

The brain was found generally gorged with blood, and the ventricles containing a good deal of serum. In above 150 cases in which the spinal marrow was examined, there was found a good deal of serum and great vascularity of the membranes. The lungs were found gorged with dark viscid oily-looking blood. Clots of fibrine were found in the cavities of the heart, and the larger blood-vessels were often lined with a membraniform sheath. The pneumo-gastric, phrenic, and splanchnic nerves were often found very vascular. The state of the intestines and other abdominal viscera is minutely described. There were marked morbid alterations in all; but, we believe, none that had not been previously observed.

Dr. Mackintosh advances nothing very satisfactory on the pathology of cholera. Like most other enquirers, his attention is arrested by the morbid condition of the blood, and he justly attributes an important part to it in the production of the symptoms. He clearly shows that the phenomena are very far from being explicable by the common notion of "a loss of balance in the circulation," as it is termed, or by congestion; and indeed disproves the existence of such pathological states. The following observation is partly the statement of a fact, and partly a pathological conjecture: it throws no new light on the subject.

"This thick blood, after finding its way into the arterial capillaries, cannot easily escape, owing to its viscidity. This is one cause of the slow motion of the blood. In many parts these small vessels give way, and ecchymosis is the consequence. This appearance has been seen in every organ of the body. It is not unreasonable to suppose that the blood becomes viscid by the abstraction of the serum, and that this is effected by the copious watery discharge from the stomach and bowels. If this view be correct, it will enable us to apply the doctrines by which Boerhaave attempted ineffectually to explain the pathology of inflammation to cholera Asiatica." (P. 355.)

We are sorry to say that Dr. Mackintosh is able to add but little to our previous knowledge, or rather ignorance, of the proper treatment of cholera. In the stage of collapse, he condemns bloodletting, purgatives, rubefacients, and seems to have derived little benefit from any other means: "we fairly tried all the remedies recommended, but observed no advantage from a large majority of them. Thus Stevens's saline solution, which it was stated had operated like magic elsewhere, was tried and laid aside."

"It was not found serviceable in any one case, and was injurious in many, by exciting vomiting and purging. The oxide of bismuth and nitrous acid were prescribed according to the directions received; but we never could discover any advantage from their use, although they were less injurious than most of the other remedies." (P. 362.)

But the most interesting part of this article is that which details the administration and effects of saline injections into the veins, a practice which seems to have been carried to a greater extent by Dr. Mackintosh than by any other physician. One hundred and fifty-six patients in all were injected in the Drummond-street Hospital, and of these twenty-five recovered. This seems but a small proportion, but it is satisfactory to know that it is *double* the proportion of patients who recovered from a similar state of collapse without the use of injections; and Dr. Mackintosh afterwards states that "not one of the patients operated on had a chance of recovery by any other means." "The substances injected were in the following proportions: muriate of soda, ℥ss.; bicarbonate of soda, ℈iv.;* water, lb. x. The temperature was from 106 to 120°. . . . . The good effects of the injection were rapid in proportion to the heat of the solution, but patients could not bear a higher temperature than that above mentioned." (P. 365.) It seems to have been of great importance to have the solution carefully prepared and cautiously administered; and we refer the reader for minute directions on these points to the work itself. Half an hour was consumed in the gradual introduction of the ten pounds of liquid.

"It was wonderful (says Dr. Mackintosh) to witness the effects speedily produced by the injection. These I shall now state under the following heads:—

"1st. *On the Pulse.* It is remarkable how speedily the injection affects the pulse, making it perceptible after it had ceased to be felt at the wrist. By the time four ounces were introduced, the pulse could generally be distinctly counted; and, when about three pounds were introduced, it became a tolerably good one, although it might be still feeble, and perhaps rapid.

"2d. *On the Cramps.* The effect on this symptom was quite remarkable: they generally ceased as soon as the pulse became good, and seldom troubled the patient again. Many cases that appeared to us hopeless, from age and the ravages of previous disease, were injected solely with a view to mitigate the sufferings of the patients produced by cramps.

"3d. *On the Temperature of the Body.* The effect on the animal heat is also almost instantaneous: the body, which could not previously be heated, now becomes warm, and, instead of a cold, damp exudation on the surface, there is a gentle and genial moisture.

"4th. *On the Respiration, &c.* The respiration, however weak previously, soon became stronger. It sometimes happened, when about four pounds of the injection were introduced, that the respiration became rather laborious, which generally gave way after more fluid was thrown into the system. The voice, which had been whispering, now became quite natural.

"5th. *On the Countenance.* In proportion as the pulse and the temperature were restored, so did the countenance improve. The eye, from being sunk, became prominent; the shrinking of the features, and the dark colour of the face and of the body, generally disappeared. The expression, in fact, become animated, and the mind lively.

"6th. *The restlessness and uneasy feelings* vanished. The despondency, vertigo, tinnitus aurium, præcordial oppression, gave way to pleasurable feelings; and I have not unfrequently seen patients sit up in bed immediately after the operation, in perfect possession of themselves, and speak with joy on the sudden transition from agony and death to happiness and life.

"7th. *Thirst,* however urgent it might have been previous to the operation, soon ceased after its commencement.

"8th. *The secretion of urine,* in general, soon returned after the injection; but in

* These proportions were afterwards doubled.

this we were more frequently disappointed than in any of the other favorable symptoms.

"9th. *The period of death* was undoubtedly postponed, sometimes for hours, more frequently for days, and sometimes even for weeks; and in some cases a perfect recovery took place." (P. 367.)

These effects are, as Dr. Mackintosh states, truly wonderful; and, although the practice may be, in its curative results, much less important than could be desired, it must certainly be looked upon with triumph and pride as one decided step towards enlarging the boundaries of our art. There is something particularly striking, and surely not a little affecting, in the instantaneous resuscitation of these persons from the very verge of the grave; and the contemplation of such things, and of the feelings of those who witnessed them, and whose hands were the instruments by which they were brought about, is sufficient to reconcile us to many of the evils and vexations with which the practice of medicine is so frequently associated. So far from being dissatisfied with the results of this practice, Dr. Mackintosh assures us that he will follow it up still more vigorously should occasion offer.

"Should I ever have charge of cholera patients again, I shall, profiting by the experience I now possess, use the saline solution at an earlier period of the stage of collapse,—nay, at its commencement,—in order to lessen the thickness of the blood before organic mischief is done, and to prevent the formation of the fibrinous clots so frequently, nay, almost invariably, found in the right side of the heart, extending into the branches of the pulmonary artery, also in the great venous channels in the head. It appeared to all who watched the symptoms, and witnessed the post-mortem examinations, that these plugs were formed during the progress of the stage of collapse, and not after death." (P. 371.)

We ought here to close our notice of Dr. Mackintosh's work, as we have already far overstepped the limits we had traced to ourselves. Were we to notice all that remains of novelty and interest in it, we should fill many more pages than we have yet done. We cannot, however, conclude without briefly adverting to a practice with which Dr. M.'s name is as indissolubly associated as with that of bleeding in intermittent fever: we allude to the treatment of Dysmenorrhœa by the mechanical dilatation of the os uteri. So long back as 1823, Dr. M. first conceived the idea that this most distressing and untractable disease might, in certain cases, depend on a disproportionate smallness of the os uteri; and a few years afterwards he put his opinion to the test of experience by dilating the orifice by means of bougies. Since that time he has employed this practice in *twenty-seven* cases, mostly after the failure of other means, and he has succeeded in *twenty-four* of these in effecting a complete cure. None of the women operated on had suffered from the disease for a shorter period than two years, some for three or four, and others for ten. Of the patients successfully treated, two-thirds were married, and of these about two-thirds (eleven) have since had children, although some of them had been married for several years (one woman as many as seven,) without ever becoming pregnant. These facts speak sufficiently for themselves, and clearly demonstrate the value of this mode of treatment. We are far from believing that a contracted state of the os uteri is the general, or even the most common cause of dysmenorrhœa; and indeed Dr. Mackintosh himself only considers it as one of the causes; but we think it is satisfactorily proved that, in many cases of dysmenorrhœa, such a state of parts does exist, and that the disease to which it gives rise is curable by means calculated to alter its mechanical relations. It is, to be sure, possible to explain the results on another principle, by considering the operation of the bougie to be rather dynamical or sympathetical than purely mechanical; but still, even on this theory, the merits of the practice are equally conspicuous. We are well aware that in this country there are innumerable objections to the general employment of such a mode of treatment, even in cases where it is particularly indicated; and some of these objections, we confess, we trust never to see removed; but every practitioner of experience must have met with cases where it would seem that it might have been had recourse to with little difficulty, and with every prospect of success; and imaginary relations will suggest themselves in which it might be of an infinite value, independently of its power to relieve bodily suffering.

"The instruments employed to produce the dilatation are the common metallic bougies, of different sizes, from that of the ordinary small silver probe to No. 14. The operation is performed (the patient lying in the position in which women are usually delivered in this country,) by introducing the index-finger of the left hand,) till it reaches the os uteri, for the purpose of directing the instrument to the part, which is then to be gently insinuated by a rotatory motion, till it arrives at the fundus of the uterus. Much force ought not to be employed, and little or no pain is produced by the operation. The unpleasant consequences which sometimes take place in treating stricture of the urethra by similar means,—viz. shivering, followed by fever,—occurred in two instances: the fever, however, was slight, and soon terminated by copious perspiration; and in these some days were allowed to elapse before the instrument was again used." (P. 434.)

We conclude by once more earnestly recommending these volumes to the attention of our readers. We advise the author, in his next edition, besides other additions already suggested, to append, in imitation of the American editor, a copious index to the work.

---

